# Profile of Transepidermal Water Loss (TEWL), Skin Hydration, and Skin Acidity (pH) in Indonesian Batik Workers

**DOI:** 10.1155/2022/7014004

**Published:** 2022-09-02

**Authors:** Cita Rosita Sigit Prakoeswa, Damayanti, Sylvia Anggraeni, Menul Ayu Umborowati, Sri Awalia Febriana, Katharina Oginawati, Ikeu Tanziha

**Affiliations:** ^1^Department of Dermatology and Venereology, Faculty of Medicine Universitas Airlangga, Dr. Soetomo General Academic Hospital, Surabaya, Indonesia; ^2^Department of Dermatology and Venereology, Faculty of Medicine, Public Health and Nursing Universitas Gadjah Mada, Dr. Sardjito Hospital, Yogyakarta, Indonesia; ^3^Faculty of Civil and Environmental Engineering, Bandung Institute of Technology, Bandung, Indonesia; ^4^Bogor Agricultural University, Bogor, Indonesia

## Abstract

Chemical substances used during batik processing may affect the physiological function of the batik worker's skin barrier. This study assessed the level of transepidermal water loss (TEWL), skin hydration, and skin acidity in 61 batik workers from the batik center in Paseseh village, Tanjung Bumi subdistrict, Madura Island, Indonesia. Forty-five batik workers involved in dry work including drawing patterns on the cloth with wax and sixteen batik workers involved in wet work including dyeing the cloth with a dye bath were included in this study. The mean TEWL level in the dry work section was 59.87 ± 11.94 g/m^2^/h on the palmar and 29.00 ± 13.09 g/m^2^/h on the dorsal side of the hand, while the mean TEWL in the wet work section were 47.39 ± 9.66 g/m^2^/h on the palmar and 37.07 ± 10.00 g/m^2^/h on the dorsal side of the hand. The mean skin hydration level in the dry work section was 49.80 ± 19.16 arbitrary units (a.u.) for the palmar side and 52.77 ± 16.21 a.u. for the dorsal side of the hand, while the mean levels of skin hydration in the wet work section were 47 ± 12.73 a.u. and 62.94 ± 10.09 a.u. for palmar and dorsal side, respectively. The mean levels of skin acidity in the dry work section were 5.45 ± 0.19 for the palmar side and 5.30 ± 0.20 for the dorsal side of the hand, while the wet work section had 5.30 ± 0.19 and 5.10 ± 0.19 for the palmar and dorsal side of the hand, respectively. The TEWL levels were found to be higher on the palmar side of the hand in both the dry work and wet work sections, which was consistent with the measurement of skin hydration levels that were lower on the palmar side of the hand. The mean skin pH levels for both work sections were considered within the normal range.

## 1. Introduction

Batik is a work of art that has been preserved well since the era of kingdoms in Indonesia back in the thirteenth century [1]. Batik has been recognized internationally after entering the UNESCO Representative List of the Intangible Cultural Heritage of Humanity on October 2, 2009. On the development and expansion of batik tradition throughout the centuries, various batik centers have grown rapidly in many cities, especially in Java Island such as Yogyakarta, Pekalongan, Surakarta, Mojokerto, and Tulungagung. Batik motifs represent the cultural norms and values of the people where the batik is produced. Therefore, each of the batik centers has its own unique characteristic which reflects the people and their local culture [1–3].

Aside from Java Island, batik centers on other islands in Indonesia were also developed and became well known throughout the country, including batik centers in Madura Island. Batik products created in Madura Island have distinct characteristics as well. The characteristics of batik products from Madura Island are the choice of colors, which are mostly bright and eye-catching such as red, yellow, blue, and green, the motifs which use floral or animal patterns, and the specific way of dyeing that use a kind of earthenware barrel to soak the cloth. The product of the latter method is known as “*batik gentongan*” which is developed in the Tanjung Bumi subdistrict, located in Bangkalan district, Madura Island [2, 4].

Producing batik cloth requires many steps, and each step is usually done by a different worker [1]. Batik workers who are involved in dry or wet work can be exposed to chemical substances that may affect their health, specifically the skin of the hands that may be exposed directly to irritative or allergenic agents including wax, dyes, solvent, and other substances used in the batik process [5, 6]. The skin that has been exposed to irritative or allergenic chemical substances may suffer from skin barrier function impairment [3, 7]. The symptoms of disrupted skin barrier such as dry skin or feeling tight on the skin may be overlooked when there is no visible deformity of the skin [8]. Consequently, this can lead to further skin inflammation or infection [7]. Batik workers should be given adequate education on how to prevent skin barrier impairment, such as limiting exposure to harmful substances, wearing personal protective equipment, and using emollients on a regular basis to preserve the barrier function and safe working environment [9].

## 2. Materials and Methods

### 2.1. Study Design

This descriptive study involved sixty-one batik workers from the batik center in Paseseh village, Tanjung Bumi subdistrict, Madura Island, Indonesia. Forty-five of the workers were assigned to dry work including drawing patterns on the cloth with wax, while sixteen workers were assigned to wet work including dyeing the cloth with dye baths, and soaking, boiling, or washing the cloth. The inclusion criteria for this study were active batik workers aged 17 years old or above and who had agreed to participate in the study. Batik workers with current dermatitis symptoms or who had any systemic disease were excluded. The study was to assess physiological skin function through transepidermal water loss (TEWL) level, skin hydration level, and skin acidity (pH) level of the hand skin of batik workers. All participants were identified and went through a skin examination. The measurement for TEWL, skin hydration, and skin acidity were assessed on the palmar side and dorsal side of the hands. The measuring tools were Courage-Khazaka® Cutometer MP-580 that consists of a Tewameter to measure the TEWL level, Corneometer to measure stratum corneum hydration level, and a pH meter to measure the skin acidity. These examinations were done consecutively during the same event. The measurements were done 3 times consecutively on both the palmar side and dorsal side, and then the data were recorded and calculated for mean values.

### 2.2. Statistical Methods

The data were analyzed with descriptive and analytical statistics using SPSS software (ver.26, IBM Corp., USA). The analytical statistics used in this study were independent samples *t*-tests and Pearson correlations because the data showed normal distributions. Significant statistical differences were determined with a *p* value below 0.05.

### 2.3. Ethics Statement

The ethical clearance of this study was approved by the Ethical Committee of Dr. Soetomo General Academic Hospital, Surabaya, Indonesia (1678/KEPK/XI/2019).

## 3. Results

This study involved a total of 61 batik workers consisting of 2 male and 59 female workers. The participants were aged between 17 and 64 years old with a mean age of 35.92 ± 10.77. The subjects were also identified by their work sections, which were dry work and wet work. All 45 workers that had been assigned to dry work were female while 16 workers that were assigned to wet work consisted of 2 males and 14 females (Table 1).

The assessment of skin function with the measurement of transepidermal water loss (TEWL) was conducted for all subjects from both work sections.  Table 2 shows that the mean values for subjects in the dry work section were 59.87 ± 11.94 g/m^2^/h on the palmar and 29.00 ± 13.09 g/m^2^/h on the dorsal side of the hand. The mean values for subjects in the wet work section were 47.39 ± 9.66 g/m^2^/h on the palmar and 37.07 ± 10.00 g/m^2^/h on the dorsal side of the hand. The lowest level of TEWL was found on a subject in the dry work section with 13.10 g/m^2^/h measured from the dorsal of the hand, and the highest level of TEWL was also found on a subject in the dry work section with 85.10 g/m^2^/h measured from the palmar of the hand. The mean TEWL for both work sections were higher on the palmar than that on the dorsal side of the hand.

Table 3 shows the level of skin hydration measured with the Corneometer for subjects in both the dry work and wet work sections. The measurement was also done on the palmar and dorsal sides of the hands. The mean levels of skin hydration for subjects in the dry work section were 49.80 ± 19.16 arbitrary units (a.u.) for the palmar side of the hand and 52.77 ± 16.21 a.u. for the dorsal side of the hand. The subjects in the wet work section had a mean level of skin hydration of 47 ± 12.73 a.u. and 62.94 ± 10.09 a.u. for the palmar and dorsal side, respectively. The lowest and highest level of skin hydration both were found for subjects in the dry work section with 13.45 a.u. measured from the palmar side as the lowest level and 109.75 a.u. measured from the dorsal side of the hand as the highest level. Meanwhile, the mean levels of skin hydration for both work sections were 49.06 ± 17.64 a.u. for the palmar side and 55.44 ± 15.45 a.u. for the dorsal side of the hand.

According to the interpretation of results from the instruction manual of the Corneometer [10], a total of 42 out of 61 subjects (68.8%) had sufficiently hydrated skin on their palm, which consisted of 30 subjects in the dry work section and 12 subjects in the wet work section. The subjects who had sufficiently hydrated skin on the dorsal side of the hand were 50 out of 61 subjects (82%) and consisted of 35 subjects in the dry work section and 15 subjects in the wet work section. In total, there were 7 subjects (11.5%) who had dry skin on their palm consisting of 6 subjects in the dry work section and 1 subject in the wet work section. A total of 10 subjects (16.4%) had dry skin on the dorsal side of the hand, consisting of 9 subjects in the dry work section and 1 subject in the wet work section. There was a total of 12 subjects (19.7%) who had very dry skin on the palmar including 9 subjects in the dry work section and 3 subjects in the wet work section. For the dorsal side of the hand, there was 1 subject who was categorized as very dry from the dry work section.

The measurement of skin acidity was conducted for all of the subjects in both work sections. The measurement was done using a pH meter on the palmar and dorsal sides of the hands.  Table 4 shows the pH levels measured for this study. The mean levels for subjects in the dry work section were 5.45 ± 0.192 for the palmar side of the hand and 5.30 ± 0.204 for the dorsal side of the hand. The mean levels for subjects in the wet work section were 5.30 ± 0.193 and 5.10 ± 0.195 for the palmar and dorsal side of the hand, respectively. The lowest skin pH level was found on a subject in the wet work section, with 4.80 measured from the dorsal side of the hand and the highest skin pH level was found on a subject in the dry work section with 5.95 measured from the palmar side of the hand. The mean skin pH levels for both work sections recorded in this study were 5.41 ± 0.202 for the palmar side and 5.24 ± 0.219 for the dorsal side of the hand.

## 4. Discussion

There are several conditions that can affect TEWL levels including endogenous, exogenous, environmental, and instrumentation factors. Endogenous factors can include age, gender, anatomical location, ethnicity, or skin condition/health. Exogenous factors can include exposure to detergents, wet work, occlusion, ingestion of caffeine, or smoking. The environmental factors include temperature, humidity, sunlight, and season. The instrumentation or measurement factor can include the type of instrument used for the study, calibration, and how the instrument was used for the measurement [11, 12]. The measurements of TEWL in this study showed that the mean TEWL levels were higher on the palmar side than the dorsal side of the hand for both the dry work and wet work sections (*p* < 0.001; mean difference = 25.478; 95% confidence interval or CI = 20.929–30.027). This could indicate that the palmar side of the hand had been in contact with surfaces and substances for more frequency or longer duration during work rather than the dorsal side, causing the increased TEWL on the palmar. There is a lack of study regarding the normal TEWL values in healthy skin [12]. A study reported a similar result of TEWL measurement on subjects with various occupations, such as the fish processing industry, metal workers, gut cleaners, nurses, and office workers. In the study, the volar side of the tip of the 3rd finger and the palm had higher TEWL compared to the dorsal side [13]. Measurement of TEWL on subjects without heavy manual work or wet work was also reported to have a higher mean TEWL on the palmar side than that on the dorsal side of the hand [14]. However, both studies did not report the comparative analysis between the palmar and dorsal sides of the hand.

**Figure 1:**
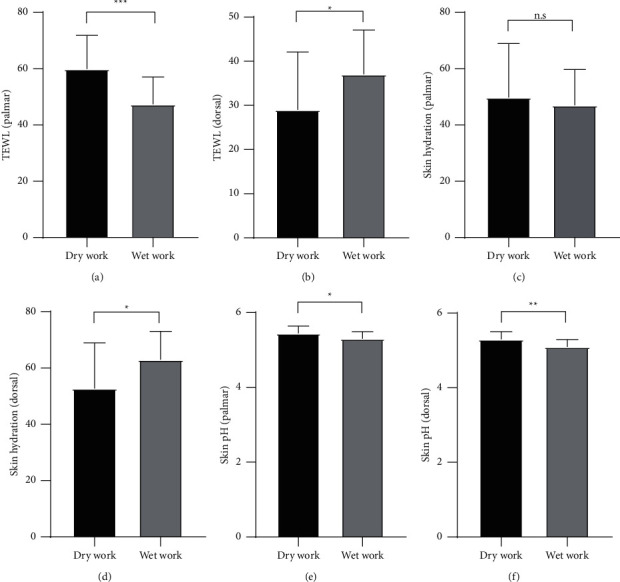
TEWL, skin hydration, and skin pH analyses of the dry and wet work sections. TEWL levels on the palmar side (a) were higher in both groups than that on the dorsal side of the hand (b) although TEWL levels in the wet work section were higher on the dorsal side rather than the palmar side. Consistent with TEWL levels, the skin hydration levels on the palmar side (c) were lower than that on the dorsal side (d) and in the wet work section, the dorsal skin hydration levels were significantly higher than the dry work section. The skin pH on the palmar side (e) and dorsal side (f) were significantly higher in the dry work section. The data are displayed as mean ± SD. ^*∗*^*p* < 0.05. ^*∗∗*^*p* < 0.01. ^*∗∗∗*^*p* < 0.001. n.s not significant.

There is a possibility that age was also a factor affecting the measurement results in this study. However, after analyzing the data on the worker's age and TEWL in this study, we found that there was no correlation between the two (palmar *p*=0.113, dorsal *p*=0.134). The other factor that could affect TEWL measurement is the work section. It is expected to be exposed to more chemical substances in wet work, potentially resulting in an increased TEWL level and drier skin [15], but the exposure to substances such as wax for a prolonged time in the dry work section could also lead to disruption of the skin barrier. In this study, we found that the wet work section had lower mean TEWL than the dry work section on the palmar side of the hand but had higher TEWL levels than the dry work section on the dorsal side of the hand. The data showed statistically significant differences between the mean TEWL for dry work and wet work on the palmar side (*p* < 0.001; mean difference = 12.479; 95% CI = 5.838–19.120) and on the dorsal side of the hand (*p*=0.029; mean difference = 8.070; 95% CI = 0.860–15.279) (Figures 1(a) and 1(b)). This finding needs further study with more focus on the correlation between the work section and the TEWL level.

The mean level of skin hydration in the dry work section and wet work section were lower for the palmar side of the hand. This was consistent with the mean TEWL levels for both work sections which were higher on the palmar side indicating more water loss; therefore, the skin hydration was lower than on the dorsal side of the hand. The disruption of the skin barrier because of irritants can lead to increased TEWL levels and disturb the water balance in the stratum corneum through the changes in lipid components and the level of natural moisturizing factor (NMF). This condition can cause decreases in stratum corneum hydration which may lead to dry skin and scaling of the skin [7]. According to some studies, the correlation between skin hydration and TEWL was found to be negative, which means that an increase in skin hydration will decrease the TEWL level [13, 16]. However, the correlation between electrical recordings of skin hydration and TEWL can be positive and linear, negative and linear, or scattered, depending on the study, the condition, and the location being studied [17].

The skin hydration measurements may also be influenced by similar factors affecting TEWL levels such as individual, exposure, environmental, or instrumental factors. From the Corneometer instruction manual, the skin hydration level measured in standard working conditions (*T* = 20–22^o^C and humidity 40–60%) is classified into 3 levels, which are very dry (< 30 a.u.), dry (30–40 a.u.) and sufficiently hydrated (> 40 a.u.) [10]. After categorizing the results, it was found that there were 12 (19.7%) workers who had very dry skin based on the results of the palmar skin hydration, whereas there was only 1 worker (1.6%) who had very dry skin on the dorsal side of the hand. There were also more workers who had sufficiently hydrated skin on the dorsal side of the hands for both work sections, indicating that the hydration on the dorsal side was better than on the palmar side which probably had been in contact more with chemical substances during batik processing. The mean skin hydration on the palmar and dorsal side of the hand had a significant difference (*p*=0.036; mean difference = 6.376; 95% CI = 0.432–12.321). The measurement of skin hydration on the 3rd finger of subjects from various occupations showed higher skin hydration on the dorsal side compared to the volar side, according to a study [13]. The result of skin hydration measurement can be affected by the location or skin area being measured and the condition of the study [17]. There was no significant difference in mean palmar skin hydration between dry work and wet work (*p*=0.517; mean difference = 2.794; 95% CI = –7.542–13.130) while the mean dorsal skin hydration between both work sections showed a significant difference (*p*=0.022; mean difference = 10.164; 95% CI = 1.486–18.841) (Figure 1(c) and 1(d)). These results should be investigated further regarding whether the work section had any influence on the skin hydration level.

In some studies, the normal pH range of the skin surface was more acidic [18–20]. Measurements ranged from 4.1 to 5.8 (95% CI), with some differences depending on anatomical body parts such as the face, trunk, and extremities [18, 21]. There are the same factors that can influence skin acidity as in TEWL and skin hydration [12, 20]. In this study, the mean pH of the workers from both the wet and dry work sections was considered within the normal range. But there were also some subjects in the dry work section who had slightly increased pH as the maximum results reached pH 5.95 and 5.82 for the palmar and dorsal side, respectively. The dry work section had higher means skin pH for the palmar and dorsal side of the hands, which could indicate that the exposure to wax and dyes or other substances in the dry work section might affect these results. The mean skin pH level of both work sections had significant differences on palmar (*p*=0.011; mean difference = 0.148; 95% CI = 0.036–0.260) and dorsal side of the hands (*p*=0.001; mean difference = 0.201; 95% CI = 0.084–0.319) (Figures 1(e) and 1(f)). The comparison of skin pH levels on the palmar and dorsal side also had a significant difference (*p* < 0.001; mean difference = 0.165; 95% CI = 0.090–0.241). Other factors might have contributed to the increased pH level of the skin in this study, such as age or washing hands with water and soap prior to measurement. One study showed that washing hands with the tap water could increase the skin pH level to about +1.0 unit, and the use of soap which had a pH of around 9.5 made pH shift on the skin up to +1.7 when measured right after a normal hand washing procedure [22]. Higher skin pH levels may affect the activity of enzymatic processes of lipid metabolism in the stratum corneum which can lead to the disruption of the skin barrier [20].

## 5. Conclusion

The TEWL levels were found to be higher on the palmar side of the hand. This indicated an increase in water loss consistent with the result of skin hydration levels for the palmar side of the hand, which were lower than the dorsal side of the hand. The mean skin pH levels of the batik workers were considered within the normal range. There might have been other factors influencing the results; therefore, further study to evaluate the correlations between the batik workers' characteristics and measurements of their skin profile is encouraged.

## Figures and Tables

**Table 1:** Demographic profile of batik workers in Paseseh village, Tanjung Bumi.

	*Work section*	
	Dry work (*n* = 45)	Wet work (*n* = 16)
Sex		
Male (%)	0 (0%)	2 (12.5%)
Female (%)	45 (100%)	14 (87.5%)
Age (years)		
Range	17–64	24–60
Mean ± SD	35.11 ± 11.26	38.19 ± 9.22
Total Mean Age ± SD	35.92 ± 10.77	

**Table 2:** TEWL profile of batik workers in Paseseh village, Tanjung Bumi.

Work section		TEWL (g/m^2^/h)	
		Palmar	Dorsal
Dry work	Mean ± SD	59.87 ± 11.94	29.00 ± 13.09
	Minimum	30.20	13.10
	Maximum	85.10	63.60

Wet work	Mean ± SD	47.39 ± 9.66	37.07 ± 10.00
	Minimum	33.30	18.95
	Maximum	64.20	54.55

Total	Mean ± SD	56.60 ± 12.59	31.12 ± 12.78
	Minimum	30.20	13.10
	Maximum	85.10	63.60

**Table 3:** Skin hydration profile of batik workers in Paseseh Village, Tanjung Bumi.

		Skin hydration	
		Palmar	Dorsal
Skin hydration profile (a.u.)			

Dry work	Mean ± SD	49.80 ± 19.16	52.77 ± 16.21
	Minimum	13.45	28.05
	Maximum	86.00	109.75

Wet work	Mean ± SD	47.00 ± 12.73	62.94 ± 10.09
	Minimum	25.18	38.35
	Maximum	69.78	77.48

Total	Mean ± SD	49.06 ± 17.64	55.44 ± 15.45
	Minimum	13.45	28.05
	Maximum	86.00	109.75

Classification of skin hydration (*n*)			
Dry work	Sufficiently hydrated	30 (66.7%)	35 (77.8%)
	Dry	6 (13.3%)	9 (20%)
	Very dry	9 (20%)	1 (2.2%)

Wet work	Sufficiently hydrated	12 (75%)	15 (93.7%)
	Dry	1 (6.3%)	1 (6.3%)
	Very dry	3 (18.7%)	0 (0%)

Total	Sufficiently hydrated	42 (68.8%)	50 (82%)
	Dry	7 (11.5%)	10 (16.4%)
	Very dry	12 (19.7%)	1 (1.6%)

**Table 4:** Skin acidity profile of batik workers in Paseseh Village, Tanjung Bumi.

Work activity		Skin pH	
		Palmar	Dorsal
Dry work	Mean ± SD	5.45 ± 0.192	5.30 ± 0.204
	Minimum	5.06	4.91
	Maximum	5.95	5.82

Wet work	Mean ± SD	5.30 ± 0.193	5.10 ± 0.195
	Minimum	4.91	4.80
	Maximum	5.64	5.58

Total	Mean ± SD	5.41 ± 0.202	5.24 ± 0.219
	Minimum	4.91	4.80
	Maximum	5.95	5.82

## Data Availability

The data used to support the findings of this study have not been made available in order to protect the privacy of participants.
